# Fulminant Ischemic Pancolitis: A Severe Variant of Ischemic Colitis

**DOI:** 10.7759/cureus.7720

**Published:** 2020-04-18

**Authors:** Anupam K Gupta, Oscar A Vazquez, Monica I Burgos, Jessica Buicko, Miguel Lopez-Viego

**Affiliations:** 1 Surgery, Charles E. Schmidt College of Medicine, Florida Atlantic University, Boca Raton, USA; 2 Internal Medicine, Universidad Autonoma de Guadalajara, Guadalajara, MEX; 3 Endocrine Surgery, Bethesda Hospital East/Florida Atlantic University, Boynton Beach, USA; 4 Surgery, Bethesda Hospital East, Boynton Beach, USA

**Keywords:** surgery, general surgery, gastroenterology, geriatrics, colitis

## Abstract

We present the case of a 75-year-old female with abdominal pain and a sudden change in mental status. She progressed rapidly with manifestations of acidosis without episodes of bloody bowel movements or diarrhea. The patient underwent emergent exploratory laparotomy, and a diagnosis of fulminant ischemic pancolitis was made with visual confirmation of infarcted colon from cecum to proximal rectum leading to subtotal colectomy and ileostomy. Postoperatively, the patient showed improved acidosis and mental status; unfortunately, over the subsequent days, the patient declined and was transferred to hospice and palliative care.

## Introduction

Ischemic colitis (IC), also known as colon ischemia, is the most common type of intestinal ischemia caused by damage to the colonic wall resulting from reduced blood flow [1]. This can vary from superficial injury of the mucosa and submucosal layer to full-thickness necrosis of the colonic wall [2]. Most instances are transient and resolve spontaneously with hospitalization and expectant management. Other cases may progress to gangrene and necrosis of the colon with resultant perforation and feculent peritonitis requiring immediate surgical intervention [3]. In terms of demographics, it mostly occurs in patients with advanced age and is more prominent among debilitated, elderly women with multiple comorbidities and/or other causes of colon hypoperfusion (i.e. medications). Its classic clinical presentation includes abdominal pain, diarrhea, and rectal bleeding with tachycardia, anemia, hypoalbuminemia, and the absence of hematochezia being factors that may indicate a poor prognosis [4-5]. Fulminant pancolitis is a rare and severe variation of IC that occurs in approximately 1% of the total cases which may be lethal due to its sudden onset with rapidly progressive course sometimes beginning with the classic IC manifestation [3]. Risk factors for this severe variant, in addition to those seen in IC, include previous aortic surgery, mesenteric artery intervention, coagulopathy, coronary artery bypass surgery, or complications of colonic surgery or colonoscopy. The underlying pathophysiology for its rapid onset is theorized to be acute non-occlusive ischemia leading to gangrenous manifestation [3,6].

## Case presentation

A 75-year-old woman with a past medical history of hypertension presented to our teaching institution with constipation for four days complicated by increasing confusion the previous 24 hours. She also reported abdominal fullness and bloating. On her arrival at the emergency department, she was tachycardic to 104 beats per minute. Blood pressure was 105/87 mmHg and she was also tachypneic at 24 breaths per minute. Significant clinical findings included altered mental status with an eye-opening response to pain, incomprehensible sounds, and flexion withdrawal to pain (Glasgow Coma Scale 8 - E2V2M4). On abdominal examination, she was distended; however, the patient could not be adequately assessed for peritonitis due to her altered mental status. Digital rectal examination showed impacted fecal matter. In view of altered mental status, the patient was immediately intubated for airway control. Her laboratory work was remarkable for a white blood cell count of 11.3 x 10^3^/µL, hemoglobin 9.5 g/dL, and platelet count 274 x 10^3^/µL. She had elevated lactate of 7 mmol/L and a creatinine 2.85 mg/dL. An arterial blood gas analysis at the time showed a pH of 7.19, pCO_2_ 52 mmHg, PO_2_ 410 mmHg, and bicarbonate 19 mEq/L. In view of mixed acidosis, fluid resuscitation was initiated.

**Figure 1:**
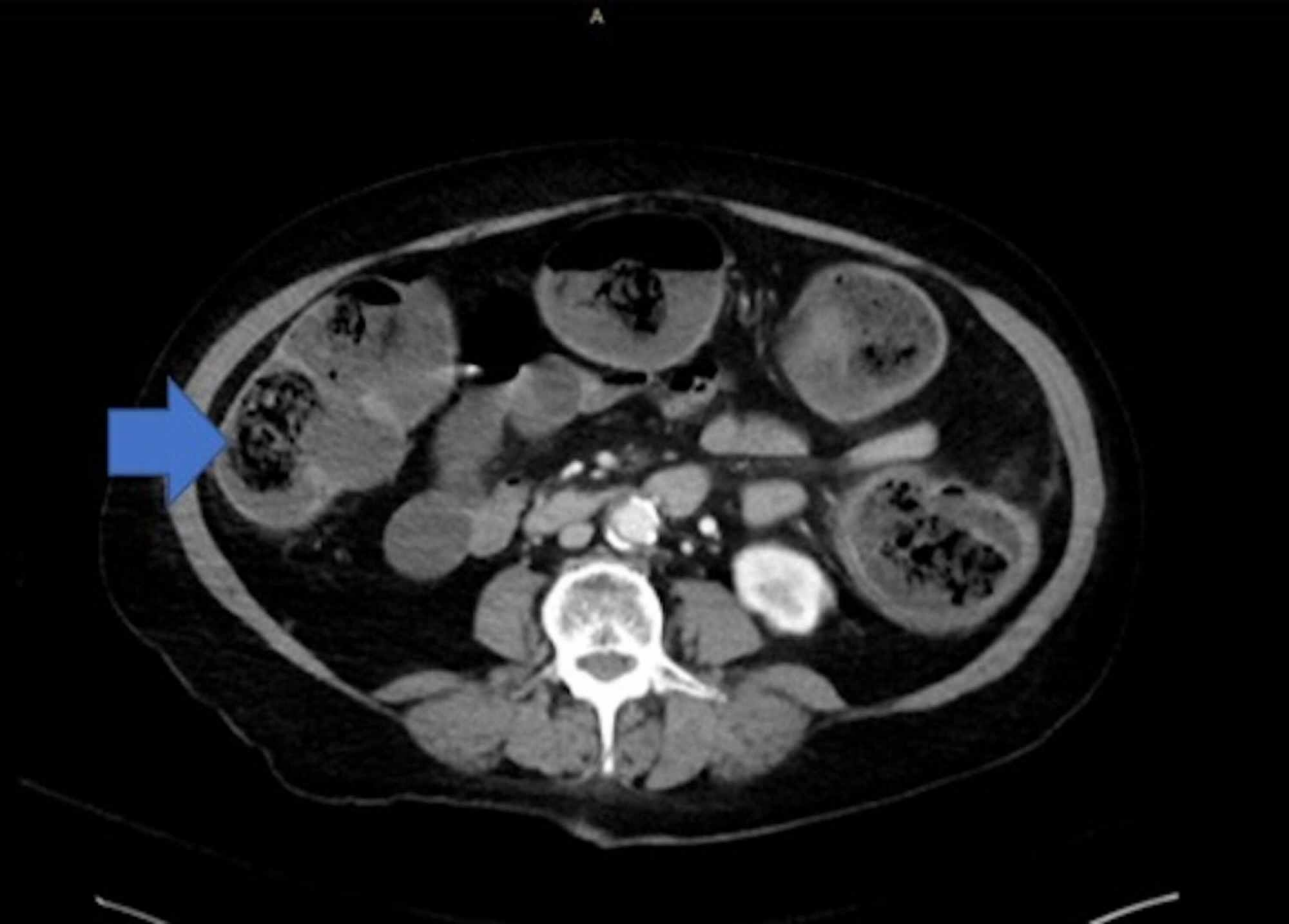
CT scan showing loaded colon with fecal matter CT, computed tomography

**Figure 2:**
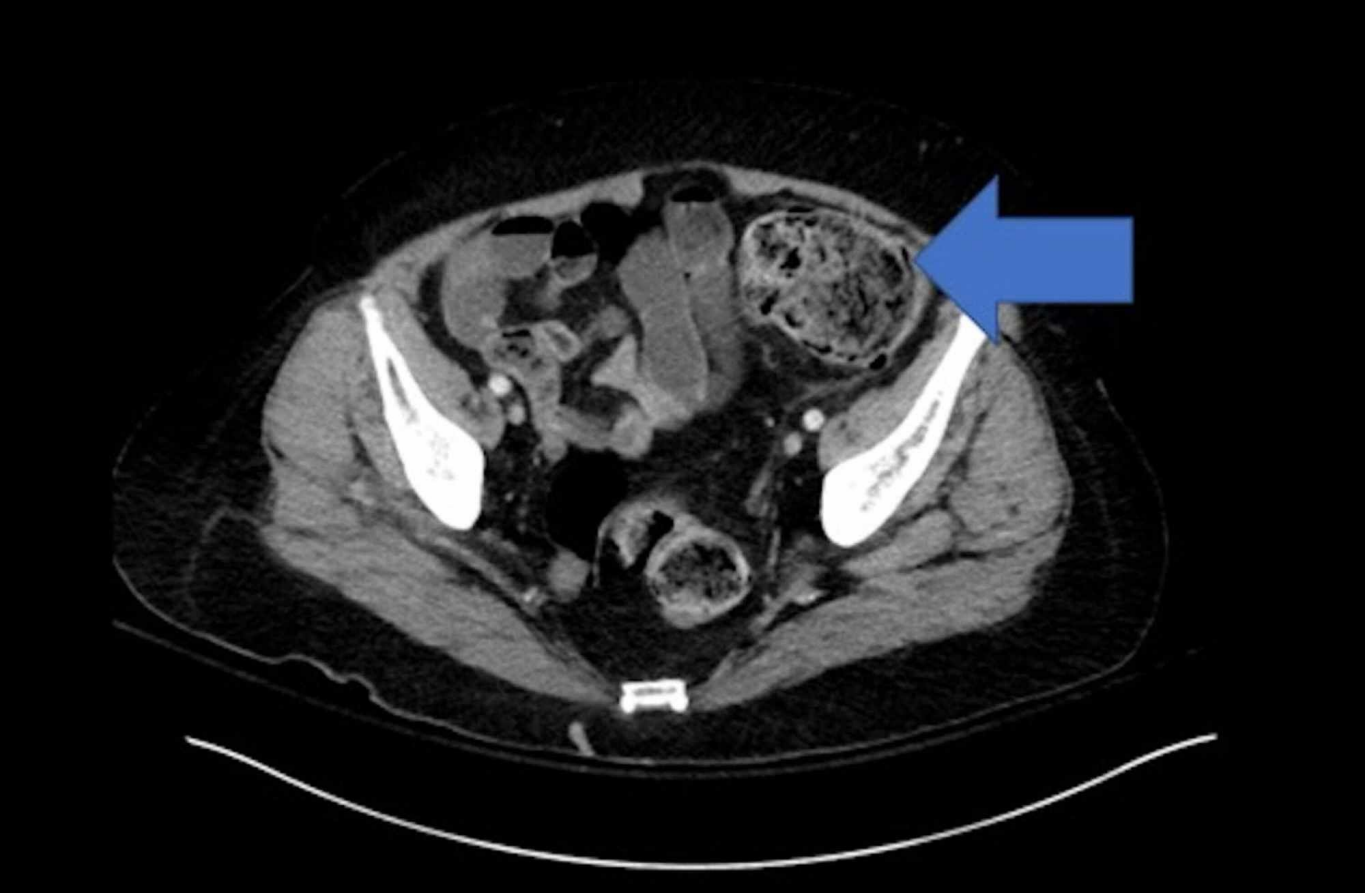
CT scan showing loaded sigmoid colon CT, computed tomography

An emergent CT scan of the abdomen and pelvis without contrast prior to intubation was positive only for loaded fecal matter in the colon (Figure 1) and a loaded sigmoid colon (Figure 2).

**Figure 3:**
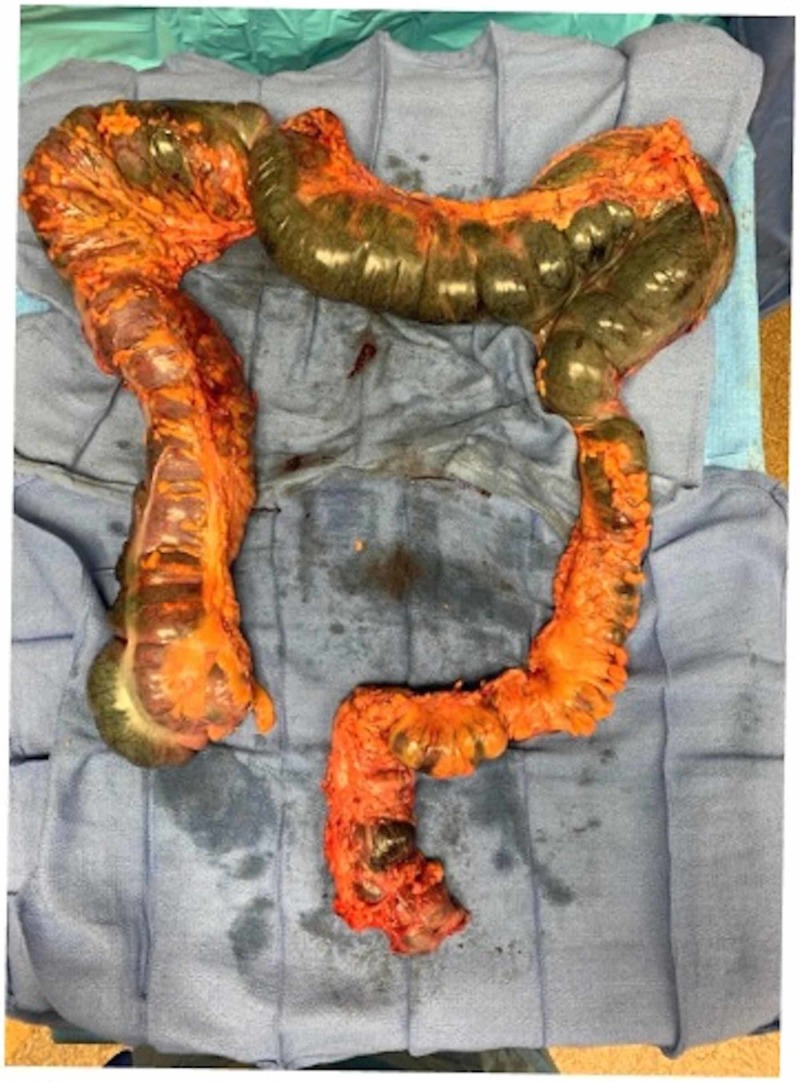
Ischemic areas extending from cecum to proximal rectum (fulminant ischemic colitis)

CT scan of the head revealed no acute pathological process. In view of acidosis and concern for ischemic bowel, the patient was emergently taken for an exploratory laparotomy which showed patchy regions of the ischemic cecum, ascending colon, transverse colon, descending colon, sigmoid colon, and proximal rectum (Figure 3). The stomach and small bowel up to ileocecal junction had a normal appearance. An intraoperative Doppler study showed patent superior and inferior mesenteric arteries and patent vasculature in the mesentery of the small bowel. The patient underwent subtotal colectomy and an end ileostomy. Postoperatively, the patient's lactic acidosis trended back to normal over the next few days, and there was a return of mental status as per the family. The pathology report of the resected colon specimen showed benign ischemic necrosis. The patient had the return of ostomy function and was tolerating tube feeds; however, due to poor physical condition, renal failure, and need for prolonged intubation, the patient’s family decided to transition to hospice and palliative care. 

## Discussion

IC can manifest with sudden cramping, mild abdominal pain in the lower left quadrant, an urgent desire to defecate, hematochezia, or melena within 24 hours of hypoperfusion. Mild-to-moderate abdominal tenderness can also be present over the involved segment of bowel. Patients with ischemia isolated to the right side of the colon present more often with lower abdominal pain than they do with rectal bleeding or bloody diarrhea [7-8]. These clinical patterns of IC are based on the degree of the histopathological damage observed in the colonic wall: reversible colopathy (submucosal or intramural bleeding), transient colitis, chronic segmental ischemia, gangrenous colitis, and, rarely, universal fulminant colitis which was seen in our patient [3,9-10]. Fulminant ischemic pancolitis may present with the classic manifestations of IC such as abdominal pain, constipation, diarrhea, hematochezia and/or vomiting, yet very little mention has been made of altered mental status as seen in our patient, though it is probable it was a sequela of her acidosis.

Laboratory studies are usually non-specific for IC and uncommon in mild ischemia and only increase with advanced and severe ischemic damage. Regardless, complete blood count, complete metabolic panel, and liver function tests are recommended to assess the physiological status of the patient [3]. Findings may include a marked leukocytosis with a predominance of immature white blood cells, an elevated hematocrit consistent with hemoconcentration, and metabolic acidosis [11]. It is also recommended that tissue injury markers should be obtained, including lactate, amylase, lactate dehydrogenase, and creatine kinase [12]. Elevated lactate, metabolic acidosis, and significant base deficit may occur in cases of severe ischemia, gangrene, and/or necrosis of the bowel wall and usually represent late, concerning signs for poor prognosis [3,13].

CT with contrast is used as the initial diagnostic test when assessing patients with non-specific abdominal pain because it may suggest the diagnosis and location, exclude other serious medical conditions, narrow the differential diagnosis possibilities, and illustrate the complications [14]. With CT, pericolic stranding with or without peritoneal fluid, thumbprinting, and initial bowel wall thickening can be appreciated in non-transmural IC. In the transmural IC, strictures may form and there is a possibility of toxic megacolon developing. CT can also help diagnose pneumatosis coli or pneumatosis intestinalis [15]. Color Doppler sonography may be useful in differentiation between inflammatory and ischemic bowel wall thickening, but it can be limited by poor sensitivity for low-flow vessel disease, bowel gas, and operator-dependent quality [16].

Regarding treatment, most cases of IC are transient and resolve spontaneously, whereas mild cases have a relatively good prognosis as they can be managed conservatively with a liquid diet, close observation, and sometimes antibiotics [17]. Increasing abdominal tenderness with guarding and rebound tenderness, fever, uncontrollable bleeding, and paralytic ileus indicate possible infarction of the colon and require urgent laparotomy and removal of the necrotic part of the colon [18]. The absence of diarrhea and hematochezia and/or hypoalbuminemia is indicative of the gangrenous form of IC [19]. More extensive ischemia in the large bowel with massive bleeding may urge for subtotal colectomy and end ileostomy. Re-anastomosis and ostomy closure are usually done after a period of four to six weeks [20]. In fulminant ischemic colitis, once identified, early operative intervention is recommended for better prognosis [3-4,6,13].

## Conclusions

Fulminant ischemic pancolitis is a rare form of IC that can involve the large bowel and spare the small bowel. Clinical manifestations may begin as IC, but later involve absence of diarrhea or hematochezia as these signs are usually associated with a poor prognosis. The severity of this variant is mostly due to its sudden onset and rapidly progressive course which must be identified early. Treatment requires emergent surgical intervention with colectomy and ileostomy.
